# Brain dysconnectivity with heart failure

**DOI:** 10.1093/braincomms/fcad103

**Published:** 2023-03-30

**Authors:** Karsten Mueller, Friederike Thiel, Birol Taskin, Frank Beutner, Andrej Teren, Vladimir K Dubovoy, Harald E Möller, Arno Villringer, Matthias L Schroeter

**Affiliations:** Max Planck Institute for Human Cognitive and Brain Sciences, Leipzig 04103, Germany; Max Planck Institute for Human Cognitive and Brain Sciences, Leipzig 04103, Germany; Clinic for Cognitive Neurology, University Hospital Leipzig, Leipzig 04103, Germany; Max Planck Institute for Human Cognitive and Brain Sciences, Leipzig 04103, Germany; Leipzig Research Center for Civilization Diseases, Leipzig 04103, Germany; Leipzig Heart Center, Leipzig 04289, Germany; Department of Cardiology and Intensive Care Medicine, University Hospital OWL of Bielefeld University, Bielefeld 33604, Germany; Max Planck Institute for Human Cognitive and Brain Sciences, Leipzig 04103, Germany; Karazin Kharkiv National University, Kharkiv 61022, Ukraine; Max Planck Institute for Human Cognitive and Brain Sciences, Leipzig 04103, Germany; Max Planck Institute for Human Cognitive and Brain Sciences, Leipzig 04103, Germany; Clinic for Cognitive Neurology, University Hospital Leipzig, Leipzig 04103, Germany; Leipzig Research Center for Civilization Diseases, Leipzig 04103, Germany; Max Planck Institute for Human Cognitive and Brain Sciences, Leipzig 04103, Germany; Clinic for Cognitive Neurology, University Hospital Leipzig, Leipzig 04103, Germany; Leipzig Research Center for Civilization Diseases, Leipzig 04103, Germany

**Keywords:** heart failure, cognitive impairment, brain connectivity, precuneus, functional magnetic resonance imaging

## Abstract

Structural brain damage associated with heart failure is well described; however, little is known about associated changes in various specific brain functions that bear immediate clinical relevance. A satisfactory pathophysiological link between heart failure and decline in cognitive function is still missing. In the present study, we aim to detect functional correlates of heart failure in terms of alterations in functional brain connectivity (quantified by functional magnetic resonance imaging) related to cognitive performance assessed by neuropsychological testing. Eighty patients were *post hoc* grouped into subjects with and without coronary artery disease. The coronary artery disease patients were further grouped as presenting with or without heart failure according to the guidelines of the European Society of Cardiology. On the basis of resting-state functional magnetic resonance imaging, brain connectivity was investigated using network centrality as well as seed-based correlation. Statistical analysis aimed at specifying centrality group differences and potential correlations between centrality and heart failure-related measures including left ventricular ejection fraction and serum concentrations of N-terminal fragment of the pro-hormone brain-type natriuretic peptide. The resulting correlation maps were then analysed using a flexible factorial model with the factors ‘heart failure’ and ‘cognitive performance’. Our core findings are: (i) A statistically significant network centrality decrease was found to be associated with heart failure primarily in the precuneus, i.e. we show a positive correlation between centrality and left ventricular ejection fraction as well as a negative correlation between centrality and N-terminal fragment of the pro-hormone brain-type natriuretic peptide. (ii) Seed-based correlation analysis showed a significant interaction between heart failure and cognitive performance related to a significant decrease of precuneus connectivity to other brain regions. We obtained these results by different analysis approaches indicating the robustness of the findings we report here. Our results suggest that the precuneus is a brain region involved in connectivity decline in patients with heart failure, possibly primarily or already at an early stage. Current models of Alzheimer’s disease—having pathophysiological risk factors in common with cerebrovascular disorders—also consider reduced precuneus connectivity as a marker of brain degeneration. Consequently, we propose that heart failure and Alzheimer’s disease exhibit partly overlapping pathophysiological paths or have common endpoints associated with a more or less severe decrease in brain connectivity. This is further supported by specific functional connectivity alterations between the precuneus and widely distributed cortical regions, particularly in patients showing reduced cognitive performance.

## Teaser

Mueller *et al*. report that heart failure is associated with reduced functional connectivity between precuneus and widely distributed brain regions. Decline in cognitive performance was specifically correlated with diminished functional brain connectivity particularly in patients with heart failure.

## Graphical Abstract

**Graphical Abstract:**
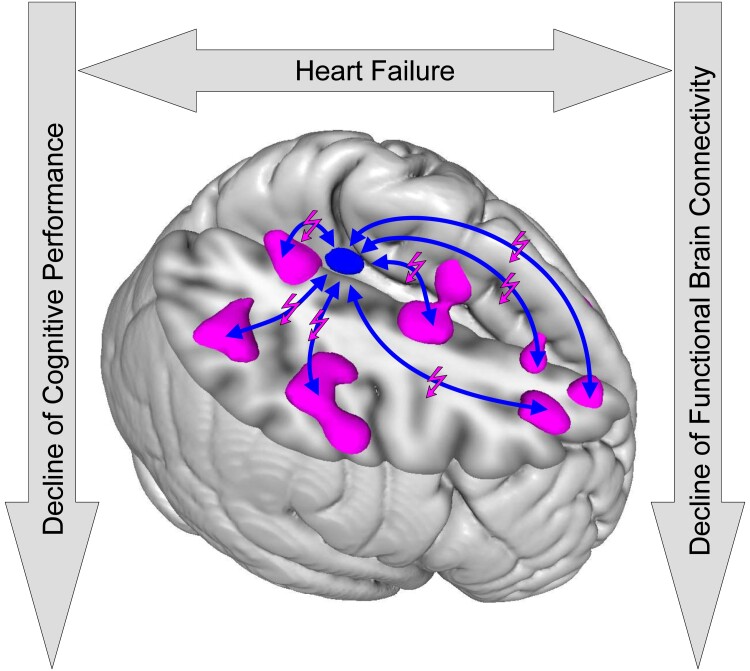

## Introduction

Due to its high metabolic activity, coupled with low-energy reserves, the brain is particularly sensitive to reductions in blood supply.^1^ Consequently, the brain is the organ that is most sensitive to transient global ischaemia after heart failure (HF).^2,3^ Besides immediate neurological complications such as mental confusion or loss of consciousness, reduced cardiac output leading to cerebral hypoperfusion may result in more or less subtle but prolonged impairment of various brain functions. Chronic HF has repeatedly been reported to be accompanied by cognitive dysfunction^4–7^ among other issues such as anxiety and mood disorders.^8–12^

The pathophysiological mechanisms altering neurocognitive performance following HF are still to be identified. Their understanding is of fundamental interest from a clinical perspective. For instance, myocardial infarction and chronic HF on the one side, and Alzheimer’s disease (AD) on the other, feature strikingly similar risk factors and comorbidities. These include arterial hypertension, hypercholesterolemia/dyslipoproteinemia (e.g. apolipoprotein E polymorphism) and metabolic syndrome (unbalanced nutrition, obesity, diabetes) as well as sedentary lifestyle, nicotine abuse and age.^13^ Consequently, a prominent research aim has been to identify distinct factors underlying apparent common aetiologies of primary neurodegenerative diseases—with its predominant representative AD—in addition to vascular/micro-angiopathic (i.e. secondary) conditions responsible for grey matter degeneration.^14–16^ Along the heart–brain axis, pathophysiological mediators that may be taken into consideration include: perturbed autoregulation of cerebral perfusion, humoral factors (catecholamines, cortisol, vasopressin and brain natriuretic peptide exerting partially antagonistic effects), metabolic consequences due to chronic hypoxaemia or oxidative stress after reperfusion, and eventually, neural cardiac reflexes that converge in the brain stem and hypothalamus.^4^ Notably, already under physiological conditions, the mutual interplay of heart and brain bears functional significance in neurocognitive processes.^17^ For instance, Al et al. recently described a considerable interference between heart action (systolic versus diastolic phase) and cortical somatosensory perceptual performance as well as cortical signalling in healthy subjects.^18^

Various cortical and sub-cortical pathologies, such as lacunar lesions or focal atrophies, are frequently encountered in patients who experience HF.^6^ In this context, we have recently explored the so-called cortical ‘grey matter density’ (GMD) changes that might represent a different type of structural damage. We performed magnetic resonance imaging (MRI) and voxel-based morphometry (VBM) in patients admitted to a local hospital exhibiting symptoms of stenocardia or myocardial infarction. Patients who had overcome HF related to coronary artery disease (CAD) subsequently developed a distributed diminution of cortical GMD as compared to CAD patients without HF.^3^ The brain regions most affected included the prefrontal cortex, temporal regions, such as the hippocampus, and most prominently, the precuneus. All of these regions represent components of attentional, memory or executive network systems that mediate complex neurocognitive functions.^19–21^ In particular, they are involved in establishing the brain’s so-called default mode network.^22–26^ On the other hand, we have demonstrated that these very same regions are involved in AD as well as its associated risk state, mild cognitive impairment (MCI).^27^ Thus, in general, these regions presumably represent crucial structures in the development of neurocognitive disorders.

Our morphometric study showed that the extent of GMD reduction was correlated with the severity of acute HF, quantified by the left ventricular ejection fraction (LVEF) and serum concentration of the N-terminal fragment of the pro-hormone brain-type natriuretic peptide (NT-proBNP), pointing to a possible direct pathophysiological link.^3^ However, counterintuitively, we did not detect any relationship between GMD and cognitive performance in these patients. This absence of evidence was suggested to result from compensatory plastic or maladaptive processes that might have evolved over time. To further investigate a potential pathophysiological link between HF and cognitive function, in the present study, and in contrast to the morphometric approach with structural MRI, we performed functional MRI (fMRI) on the very same patient cohort. Haemoglobin-oxygenation/blood-flow-dependent signal was acquired in a task-free manner using the so-called blood oxygenation level dependent (BOLD) resting-state scans. This allows a topographic map of functional brain connectivity to be computed after statistical data analysis. We aimed to detect regional alterations in functional connectivity, more specifically, non-structural brain correlates of HF that accompany changes in cognitive performance. In particular, and similar to the morphometric study, we were looking for potential, stage-dependent, correlations of functional connectivity with both initial and follow-up values of LVEF and NT-proBNP. We were interested in how regions that had been identified as having diminished cortical GMD would topographically relate to potential changes in functional connectivity. For instance, one might assume that regionally specific structural degradation of cortical tissue would result in a disturbance or rearrangement of functional connectivity within the default mode network. Finally, by performing particular statistical correlation analyses, we addressed our key question: Is there a significant dependence between changes in functional brain connectivity and cognitive performance in HF patients?

## Materials and methods

### Patient cohort

The study included a total of 80 patients from the Leipzig Heart Center (22 females; age 54.9 ± 5.3 years; mean ± SD). An illustration of the patient recruitment and demographic details are given in a preceding study.^3^ All participants provided informed written consent. The study was carried out in accordance with the Declaration of Helsinki and approved by the Ethics Committee of the University of Leipzig (ID 099-12-05032012).

Participants were grouped into patients with CAD (*N* = 58; 14 females; age 54.6 ± 5.4 years) and patients, in whom no CAD had been detected (no-CAD; *N* = 22; 8 females; age 55.7 ± 5.0 years). Patients with CAD were further divided into patients with and without HF (CAD+ and CAD−, respectively) according to the guidelines of the European Society of Cardiology.^28^ A defining criterion of the guidelines is based on the NT-proBNP serum concentration. For patients with an intermediate age, HF is likely associated with NT-proBNP level >900 pg/mL in an acute setting.^29^ Using this definition, our CAD cohort included 35 patients with HF (CAD+). Note that we excluded two patients from the CAD+ group due to lacunar/ischaemic cerebral lesions. This resulted in 33 patients in the CAD+ group (8 females; age 53.8 ± 5.8 years). HF is unlikely with NT-proBNP values <300 pg/mL in an acute setting, independent of the patient’s age.^29^ Thus, the CAD− group was defined using a threshold of maximum NT-proBNP level of 300 pg/mL. This resulted in 20 CAD− patients (4 females; age 56.1 ± 5.0 years). Three CAD patients showing acute NT-proBNP levels between 300 and 900 pg/mL (defined as ‘grey zone’ in Mueller et al.^29^) were assigned neither to the CAD+ nor to the CAD− group and were, therefore, excluded from all group comparisons.

Upon hospitalization, clinical examination was performed and para-clinical parameters were assessed in all patients. This included LVEF (by means of transthoracic echocardiography) and serum concentrations of NT-proBNP. Throughout the manuscript we will refer to these parameters as the *initial* values of LVEF and NT-proBNP. Patients were invited for a *follow-up* session after 3.5 ± 1.3 years, where LVEF and NT-proBNP were obtained again. The follow-up session also included MRI data acquisition as well as assessment of cognitive performance using standardized cognitive tests; thus, MRI and cognitive tests were only obtained in the follow-up session. Cognitive tests were performed to cover all cognitive domains including attention, learning, memory and executive function. In particular, the following cognitive tests were used: Trail making test A and B,^30^ Test battery of attentional processes (alertness, divided attention),^31^ Hamasch five-point test revised,^32^ Regensburg word fluency test,^33^ Stroop test, California verbal learning test^34^ and Rey-Osterrieth complex figure test.^35^ Individual raw values were transformed into age-matched and, if applicable, sex-matched normative values. Finally, participants were assessed with respect to normative controls using percentile ranks for each cognitive domain. Cognitive performance was obtained using the mean percentile rank across all four cognitive domains as a measure for general cognitive ability. Finally, participants were categorized into either above or below average.

### Image acquisition

Functional MRI data of the brain were obtained using a 3-T MAGNETOM Verio scanner (Siemens Healthineers, Erlangen, Germany) with a 32-channel head receive array and a T_2_*-weighted gradient-echo echo-planar imaging (EPI) sequence (repetition time, TR 2 s; echo time, TE 30 ms; flip angle 90°; pixel bandwidth 1953 Hz). The following image dimensions were used: acquisition matrix 64 × 64 pixels, 30 axial slices with a slice thickness of 4 mm (0.8 mm gap, ascending slice order), nominal image resolution 3 × 3 × 4.8 mm^3^. For each participant, 300 functional volumes were acquired resulting in a total scanning duration of 10 min. Image acquisition was performed in the so-called ‘resting-state’. That is, participants were instructed to fixate a visual crosshair and to remain still and awake, but they had no specific cognitive task to perform.

Additional *T*_1_-weighted images were acquired with a magnetization-prepared rapid gradient-echo (MPRAGE) sequence for registration and normalization purposes (TR 2300 ms; inversion time 900 ms, TE 2.98 ms, flip angle 9°; acquisition matrix 176 × 240 × 256 pixels, nominal image resolution 1 × 1 × 1 mm^3^).

### Image pre-processing

All fMRI data sets were analysed using the CONN toolbox rev. 21a^36^ and SPM12 rev. 7771^37^ (Wellcome Centre for Human Neuroimaging, University College London, UK) with MATLAB 9.12 rev 2022a (The MathWorks Inc., Natick, MA). Pre-processing was performed using the default pipeline within the CONN toolbox^36^ including realignment for motion correction, unwarping of EPI images to correct for distortions, slice-time correction and normalization to the Montreal Neurological Institute space based on the unified segmentation approach.^38^ Normalization was performed with the default settings for resampling and interpolation using a destination resolution of 2 × 2 × 2 mm^3^. Thereafter, spatial filtering was applied using a Gaussian kernel with 8-mm full width at half maximum.^39,40^ Image pre-processing also included denoising within the CONN toolbox. To correct for nuisance signal fluctuations, a regression analysis was computed using the signal from regions of cerebrospinal fluid (16 regressors) where no ‘true’ brain activation should be located, as well as the translational and rotational parameters from head movements obtained by SPM (six regressors), the scan-to-scan changes in global signal and the frame-wise displacement (FD) timeseries (two regressors), and the effect of the beginning of the resting-state measurement (two regressors). Pre-processing was finalized using detrending and despiking, and band-pass filtering with the cut-off frequencies of 0.025 and 0.2 Hz to achieve a baseline correction.

### Centrality analysis

For each patient, brain network centrality was computed within the CONN toolbox^36^ using global correlation (GCOR). GCOR represents a measure of node centrality at each voxel, characterized by the strength and sign of connectivity between a given voxel and the rest of the brain. GCOR is defined as the mean of correlation coefficients between each individual voxel and all of the voxels in the brain. We used GCOR to detect the major hubs of brain networks and differences of these hubs between different groups of patients. Furthermore, we also used GCOR to investigate potential correlations between network centrality and the LVEF and NT-proBNP biomarkers. In addition to GCOR, we computed a further centrality measure, namely eigenvector centrality (EC),^41^ using the fastECM software.^42^ To obtain the EC a similarity matrix was computed using the correlation coefficient between all resting-state fMRI time courses. In order to use a similarity matrix with non-negative elements, we added a value of one to all correlations (also implemented in the Lipsia software package^43^).

After having computed both types of centrality maps for all patients, group analyses were performed using SPM12 with a general linear model (GLM) including age, sex and body mass index (BMI) as additional nuisance regressors. Group comparisons were performed within a single model using a full-factorial design with a group factor including (i) CAD patients with HF (CAD+); (ii) CAD patients without HF (CAD−) and (iii) patients without CAD (no-CAD). After parameter estimation, subsequent statistical analysis was performed investigating the main effect of the group factor employing an *F*-test. In addition, *post hoc* group comparisons were obtained with the same model using *t*-contrasts in order to detect potential centrality differences (i) between CAD patients with and without HF (CAD+ versus CAD−); (ii) between CAD patients with HF and those without CAD (CAD+ versus no-CAD) and (iii) between all patients with and without HF (CAD+ versus ALL−). Here, the notation ALL− denotes the group of all patients showing no HF (including all patients of the CAD− and the no-CAD group). Statistical parametric maps were obtained using SPM12 with a cluster-forming threshold of *P* < 0.001.^44^ Significant clusters were obtained at *P* < 0.05 using family-wise error (FWE) correction for multiple comparisons at the cluster-level.^44–46^

In order to check for normality of the GCOR and EC values within the three groups (CAD+, CAD−, no-CAD), we performed a Shapiro-Wilk test^47,48^ at each voxel using an alpha level of 0.05 including a Bonferroni correction based on the number of resolution elements^46^ obtained from the SPM analysis.

In addition to group comparisons, we performed further GLM analyses across all participants in order to identify potential relationships between centrality measures (both GCOR and EC) and LVEF or NT-proBNP. Here, regression analyses were performed using a GLM including LVEF or NT-proBNP, respectively, as a covariate of interest, while age, sex and BMI were again used as nuisance regressors. The covariate of interest included either the initial or the follow-up measurement of LVEF or NT-proBNP, respectively. The resulting statistical parametric maps were assessed with the same procedure as for the group comparisons using a cluster-defining threshold of *P* < 0.001 and significant clusters at *P* < 0.05 using FWE-correction at the cluster-level.^44–46^

### Seed-based correlation analysis

In order to investigate functional connectivity differences associated with centrality alterations, we also performed a seed-based connectivity analysis using a seed region obtained with the centrality analysis. A map of the seed region was generated based on all voxels showing GCOR differences (*P* < 0.05 using FWE-correction at voxel-level) between patients with and without HF (CAD+ < ALL−). Thereafter, this seed region was added as a region of interest in the CONN toolbox, and, for each participant, a seed-based correlation analysis was performed using the average signal within the seed region. The individual correlation maps were then entered into a group analysis using SPM12 with a GLM implementing a full-factorial design. This model was built using two factors: HF (CAD+ versus ALL−) and cognitive performance (above versus below average). In addition, the model also included age, sex and BMI as additional nuisance covariates. Statistical analysis tested for an interaction between the factors HF and cognitive performance with respect to seed-based brain connectivity. In addition, *post hoc* tests were used to look at potential connectivity differences related to cognitive performance within the CAD+ and the ALL− group separately. Resulting statistical parametric maps were assessed with the same procedure as the centrality analysis, using a cluster-defining threshold of *P* < 0.001 and clusters significant at *P* < 0.05 using FWE-correction at the cluster-level.^44–46^

In addition to the 2 × 2 factorial design described above, a further factorial model was generated using the same two factors (HF; cognitive performance) but implementing cognitive performance as a continuous variable using the mean of the percentile ranks of the cognitive domains. The interaction between the categorial and the continuous variable (HF versus cognitive performance) was implemented with SPM12, and statistical analysis tested for an interaction between both factors with respect to seed-based brain connectivity.

### Statistical analysis including grey matter density

To investigate the relationship between functional connectivity and structural GMD differences, all statistical analyses were re-computed using GMD as an additional nuisance covariate. GMD values were extracted from the GMD images^3^ with the same mask as used in the seed-based correlation analysis described above. The GMD covariate was added to the full-factorial model as well as in all correlational analyses (with LVEF and NT-proBNP). In addition, we also included the GMD nuisance covariate in the full-factorial model with the seed-based correlation maps in order to investigate whether structural and functional changes can explain an independent portion of the variance of cognitive decline.

### Motion effects

Head motion during fMRI data acquisition can induce signal fluctuations and thus could obscure the connectivity analysis and the resulting connectivity values. This could be a particular problem if the degree of motion varied systematically between patients with and without HF, i.e. between the CAD+ and the CAD− group, or between the CAD+ and the no-CAD group. Therefore, we checked for differences in head motion between these groups using the FD values^49^ from the image pre-processing. For the entire series of 300 functional images, head motion between volumes was obtained by 299 FD values for each subject. For each subject, head motion was characterized by mean and maximum FD. Statistical analysis was performed to detect significant differences of mean and maximum FD between the three groups CAD+, CAD− and no-CAD.

### Robustness analysis

In order to check for the robustness of our results with respect to the processing pipeline, various analyses were performed using different analysis parameters. Firstly, we checked our GCOR results using a different cut-off for band-pass filtering. Our original setting using cut-off frequencies of 0.025 and 0.2 Hz was aimed at investigating brain connectivity based on the so-called low-frequency oscillations around 0.1 Hz, namely between 0.07 and 0.12 Hz.^50,51^ However, other work suggests using considerably lower frequencies up to 0.01 Hz.^52^ Therefore, we re-computed the pre-processing using a band-pass filter with a cut-off frequency of 0.01 Hz instead of 0.025 Hz. Thereafter, we re-computed all GCOR images and re-performed all statistical analyses (GCOR group comparisons with the full-factorial model and all GCOR correlation analyses).

A second additional analysis aimed at confounding effects of subject movement during image acquisition. We performed a separate analysis of the subject’s motion using the six motion parameters obtained by SPM (see section above) and additionally included them as covariates during nuisance regression. To further consider the subject’s motion in our analysis, we re-computed the pre-processing adding first-order derivatives and quadratic effects of the motion parameters resulting in 24 covariates (frequently referred to as the Friston24 model).^53^ Thereafter, we again re-computed all GCOR images and re-performed all statistical analyses (GCOR group comparisons with the full-factorial model and GCOR correlation analyses).

Note that all statistical analyses include the BMI as nuisance covariate as a major cardiovascular risk factor. To investigate the influence of further cardiovascular risk factors on the fMRI data and subsequently on functional brain connectivity, we re-performed all GCOR analyses (group comparisons with the full-factorial model and GCOR correlation analyses) using additional nuisance covariates. In particular, we added covariates for nicotine abuse, diabetes mellitus, systolic and diastolic blood pressure, and the Fazekas score^54^ controlling for white matter hyperintensities. Finally, we also added a covariate including the total intra-cranical volume obtained from the VBM analysis in a preceding study.^3^

### Visualization

Figures showing orthogonal brain slices were generated using the Mango software v4.1 (Research Imaging Institute, University of Texas Health Science Center, San Antonio) with the ‘Build Surface’ option and the ‘Cut Plane’ feature. Statistical parametric maps were imported using the ‘Add Overlay’ function. Dot plots were directly obtained from SPM12 and plotted with MATLAB.

## Results

### Normality of centrality values

In order to check for normality of the centrality values, a Shapiro-Wilk test^47,48^ was performed across all voxels of the GCOR and EC maps within all three groups of patients CAD+, CAD− and no-CAD. Significant results were obtained for very few voxels only (GCOR: <0.1% of all voxels; EC: <0.65% of all voxels). Significant voxels were randomly distributed across the image.

### Centrality group differences

**Figure 1:**
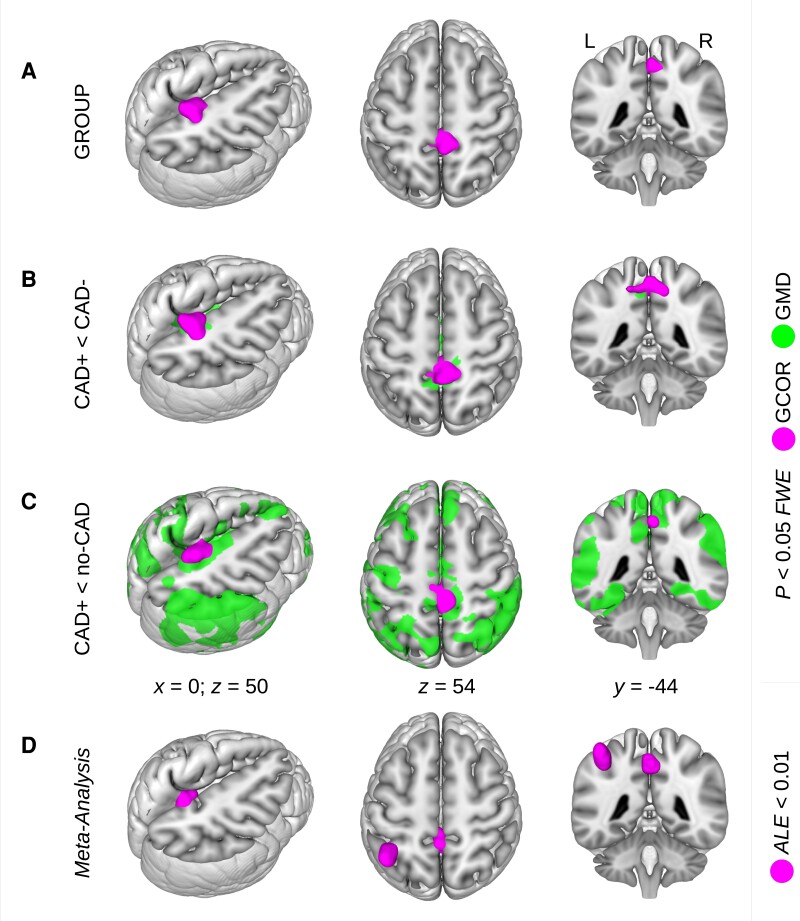
**Brain network centrality decrease in patients with heart failure.** Investigating global correlation (GCOR) of all three groups of patients with a full-factorial model including (i) patients with coronary artery disease (CAD) with heart failure (CAD+); (ii) CAD patients without heart failure (CAD−) and (iii) patients without CAD (no-CAD), we obtained a significant main effect using (**A**) an *F*-contrast across the group factor. *Post hoc* group comparisons were performed within the same model using *t*-contrasts and revealed a GCOR decrease (**B**) when comparing CAD+ with CAD− patients, and (**C**) when comparing CAD+ patients with no-CAD patients. All results were located in the precuneus but in no other brain region. Results were obtained with *P* < 0.05 using family-wise error (FWE) correction at cluster-level (see Table 1 for all comparisons including both centrality measures of GCOR and eigenvector centrality). Note, in a preceding study,^3^ a grey matter density (GMD) decrease was found with the same comparisons in the very same cohort particularly in the precuneus. (**D**) The obtained GCOR group differences are located in the same brain region within the precuneus, which was detected in a systemic and quantitative meta-analysis^27^ investigating patients converting from mild cognitive impairment to Alzheimer’s disease. ALE = activation likelihood estimation; L = left; R = right; *x, y, z* = coordinates in mm.

**Figure 2:**
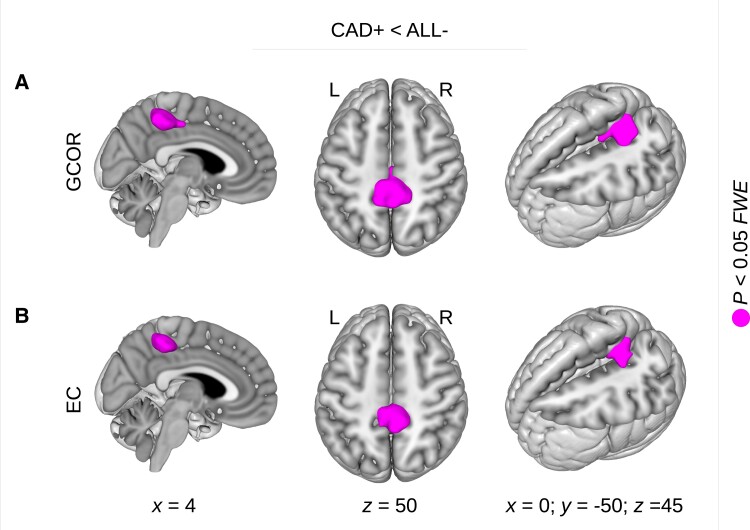
**Brain network centrality decrease in patients with heart failure.** A significant network centrality decrease was obtained when comparing patients with heart failure and coronary artery disease (CAD+) with all other patients without heart failure (ALL−). This network centrality decrease was obtained with both (**A**) global correlation (GCOR) and (**B**) eigenvector centrality (EC). With both GCOR and EC, centrality decrease was obtained in the precuneus but in no other brain region. Statistical analysis was performed using a general linear model with a full-factorial design implementing the group comparison using a *t*-contrast. Results were obtained with *P* < 0.05 using family-wise error (FWE) correction at cluster-level (see Table 1, bottom rows). L = left; R = right; *x, y, z* = coordinates in mm.

Using the full-factorial design implementing a group factor containing all centrality images of the three groups, i.e. CAD+, CAD− and no-CAD, we obtained a significant main effect with both GCOR and EC in the precuneus (*P* < 0.05, FWE-corrected; Table 1; Fig. 1A). Subsequent group comparisons within the same model gave consistent centrality differences showing a reduced GCOR and EC with HF. Comparing CAD groups with and without HF (i.e. CAD+ < CAD−), patients with HF showed a significantly diminished centrality in the precuneus (*P* < 0.05, FWE-corrected; Table 1; Fig. 1B). Interestingly, the same result was found when replacing the CAD− group with the no-CAD group (i.e. CAD+ < no-CAD; *P* < 0.05, FWE-corrected; Table 1; Fig. 1C). The consistency of the two comparisons indicates a reduced centrality with HF rather than an increase of centrality in the other groups. Comparing the CAD+ group with all non-HF participants (denoted by ALL−), we again found a significantly reduced centrality with HF in the precuneus with both GCOR and EC (*P* < 0.05, FWE-corrected; Table 1; Fig. 2). Notably the obtained group differences are located in exactly the same brain region, within the precuneus, which was detected in a previous comprehensive meta-analysis^27^ predicting conversion from MCI to AD. Note that this meta-analysis result was re-calculated using the normalization of SPM12 and visualized with Mango v4.1 (Fig. 1D).

**Table 1:** Brain network centrality decrease with heart failure*

		pFWE	*K_E_*	*F/T*	*Z*	*x*	*y*	*z*
GROUP	GCOR	**<0.001**	**404**	**17.06**	**4.76**	**4**	**−36**	**52**
				14.12	4.34	−6	−34	48
				10.82	3.77	16	−40	50
	EC	**0.015**	**227**	**15.32**	**4.52**	**6**	**−36**	**52**
				11.07	3.82	−4	−34	50
CAD+ < CAD−	GCOR	**<0.001**	**602**	**4.94**	**4.56**	**−8**	**−34**	**48**
				4.79	4.44	6	−40	52
				4.65	4.32	16	−40	50
	EC	**0.004**	**401**	**4.72**	**4.38**	**6**	**−38**	**52**
				4.40	4.12	−6	−34	50
				4.05	3.82	14	−38	50
CAD+ < no-CAD	GCOR	**0.004**	**380**	**5.09**	**4.68**	**4**	**−36**	**52**
				4.20	3.95	10	−26	46
				4.00	3.78	−8	−30	48
	EC	**0.047**	**226**	**4.82**	**4.46**	**4**	**−36**	**52**
				3.72	3.53	10	−28	48
				3.65	3.48	−8	−30	48
CAD+ < ALL−	GCOR	**<0.001**	**797**	**5.83**	**5.24**	**4**	**−36**	**52**
				5.22	4.77	−6	−34	48
				4.22	3.96	14	−34	46
	EC	**0.001**	**516**	**5.53**	**5.02**	**6**	**−36**	**52**
				4.67	4.34	−4	−34	50
				3.84	3.65	14	−34	46

*Significant network centrality differences using the measures of global correlation (GCOR) and eigenvector centrality (EC). Using a full-factorial design implementing a GROUP factor containing all centrality images of the three groups of patients (i) with coronary artery disease (CAD) with heart failure (CAD+); (ii) with CAD but without heart failure (CAD−) and (iii) with no CAD (no-CAD), we obtained a significant main effect in the precuneus with both centrality measures GCOR and EC (see upper rows). Interestingly, the same precuneus region was obtained when looking at centrality differences between CAD patients with and without heart failure (CAD+ < CAD−), and when looking at centrality differences between CAD patients with heart failure and patients without CAD (CAD+ < no-CAD). The bottom rows show a comparison of network centrality between patients with heart failure and all other patients showing no heart failure (CAD+ < ALL− where ALL− denotes all patients of the CAD− and the no-CAD group). The table shows the *F*- and *T*-maximum (respectively) of each cluster (in bold) and further local maxima more than 8 mm apart. pFWE = *P*-value with family-wise error correction at cluster-level; *K_E_* = cluster size in voxels; *x, y, z* = coordinates in mm.

We also performed tests for the inverse contrast, looking at potential centrality increases with HF. The following group comparisons were performed: CAD+ > CAD−, CAD+ > no-CAD and CAD+ > ALL−. No significant results were found for either measure of centrality.

Our robustness analyses showed only subtle changes of the results when investigating the main group effect and all group comparisons with variations of the analysis parameters; thus, we received significant GCOR differences in the same anatomical location, namely in the precuneus (see Supplementary Table 1 for using band-pass filtering with a cut-off frequency of 0.01 Hz instead of 0.025 Hz; see Supplementary Table 2 for using the Friston24 model instead of the six motion parameters and see Supplementary Table 3 using additional nuisance covariates in order to account for smoking, diabetes mellitus, systolic and diastolic blood pressure, Fazekas score and total intracranial volume).

### Centrality correlation analysis

**Figure 3:**
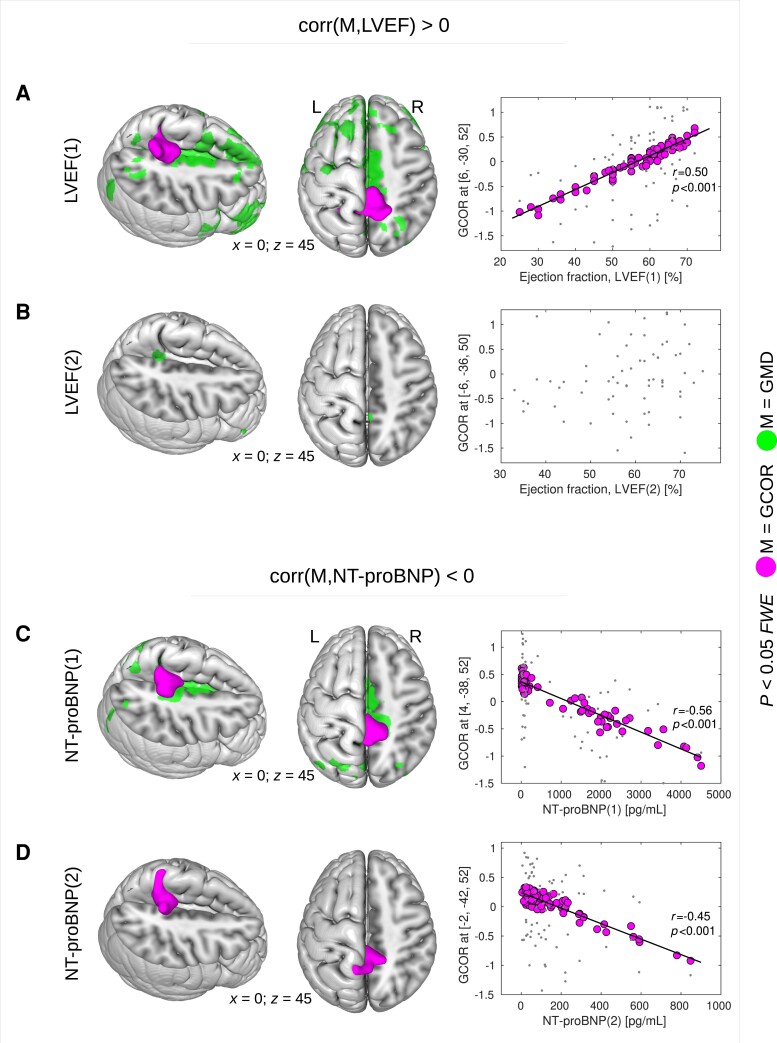
**Correlation between heart failure-related biomarkers and brain network centrality across all patients.** (**A**) A significant positive correlation was detected between the initial measurement of left ventricular ejection fraction (LVEF(1)) and global correlation (GCOR) in the precuneus. Reduced LVEF(1) values were associated with diminished GCOR. (**B**) The follow-up measurement LVEF(2) did not show any significant correlation with GCOR. (**C**) A significant negative correlation was found between the initial measurement of N-terminal fragment of brain natriuretic peptide (NT-proBNP(1)) and GCOR in the precuneus. (**D**) A significant negative correlation was also found between the follow-up measurement NT-proBNP(2) and GCOR in the same region, namely the precuneus. For both the initial and the follow-up measurements NT-proBNP(1) and NT-proBNP(2), respectively, higher serum levels were associated with diminished GCOR values. Statistical analyses were performed using a general linear model implementing a one-sample *t*-test including a *t*-contrast with the covariate of interest. Results were obtained with *P* < 0.05 using family-wise error (FWE) correction at cluster-level (see Tables 2 and 3). Note that, in a preceding study,^3^ a significant positive correlation was found between grey matter density (GMD) and both LVEF(1) and LVEF(2) in the same cohort of patients (see GMD in **A** and **B**). The same study shows also a negative correlation between GMD and NT-proBNP(1) (see GMD in **C**). L = left; *M* = image modality; R = right; *x, y, z* = coordinates in mm.

Correlation analyses were performed across all participants in order to investigate a potential relationship between brain centrality and LVEF using both the initial and the follow-up measurement. Here, we found a significant positive correlation between both network centrality measures (GCOR and EC) and the initial LVEF(1) measurement in the precuneus (*P* < 0.05, FWE-corrected; Table 2; Fig. 3A). Lower LVEF values were associated with less network centrality. Note that we did not obtain this finding when using the follow-up LVEF(2) measurement. That is, we did not find a significant correlation between LVEF(2) and network centrality, for GCOR or EC (see Fig. 3B).

**Table 2:** Correlation between heart ejection fraction and brain network centrality across all patients*

		pFWE	*K_E_*	*T*	*Z*	*x*	*y*	*z*
LVEF(1)	GCOR	**<0.001**	**1021**	**5.09**	**4.69**	**6**	**−30**	**52**
				4.72	4.39	−6	−44	50
				4.36	4.09	8	−46	56
	EC	**<0.001**	**733**	**4.74**	**4.41**	**6**	**−36**	**54**
				4.26	4.01	8	−46	56
				3.95	3.75	−6	−46	50

*Significant positive correlation between left ventricular ejection fraction (LVEF) and network centrality using the measures of global correlation (GCOR) and eigenvector centrality (EC). A significant positive correlation was obtained between the initial measurement of ejection fraction LVEF(1) and both centrality measures in the same precuneus region as obtained with the group centrality differences (see Table 1). Note that this finding was not obtained using the follow-up measurement of ejection fraction LVEF(2). The table shows the *T*-maximum of each cluster (in bold) and two further local maxima more than 8 mm apart. pFWE = *P*-value with family-wise error correction at cluster-level; *K_E_* = cluster size in voxels; *x, y, z* = coordinates in mm.

We also checked for the opposite direction of correlation, i.e. for a negative correlation between network centrality and LVEF. Here, no significant results were obtained with LVEF(1). Nevertheless, a negative correlation between centrality and the follow-up measurement LVEF(2) was found in the left prefrontal cortex when using GCOR; however, this result did not survive FWE-correction when using EC (*P* = 0.096).

In addition to LVEF, we also looked at potential correlations between centrality measures and NT-proBNP across all participants. The analysis showed a significant negative correlation between brain network centrality and NT-proBNP in the precuneus. This negative correlation was obtained with both centrality measures, GCOR and EC, and with both the initial and the follow-up measurements of NT-proBNP (*P* < 0.05, FWE-corrected; Table 3; Fig. 3C and D). Higher levels of NT-proBNP were associated with lower centrality values in the precuneus. In addition to the precuneus, we also found a significant negative correlation between EC and the follow-up measurement NT-proBNP(2) in cerebellar regions (Table 3, bottom rows). This result was not obtained when using GCOR instead of EC.

**Table 3:** Correlation between the biomarker N-terminal fragment of brain natriuretic peptide (NT-proBNP) and brain network centrality across all patients*

		pFWE	*K_E_*	*T*	*Z*	*x*	*y*	*z*
NT-proBNP(1)	GCOR	**<0.001**	**753**	**6.02**	**5.41**	**4**	**−38**	**52**
				5.16	4.75	14	−38	50
				5.11	4.71	−6	−36	48
	EC	**<0.001**	**565**	**5.85**	**5.28**	**6**	**−38**	**52**
				4.76	4.43	−4	−36	50
				3.35	3.22	−2	−46	62
NT-proBNP(2)	GCOR	**<0.001**	**656**	**4.47**	**4.19**	**−2**	**−42**	**52**
				4.21	3.97	8	−38	46
				4.21	3.97	−8	−48	74
	EC	**<0.001**	**1960**	**5.56**	**5.06**	**−10**	**−46**	**72**
				4.74	4.42	0	−38	54
				4.56	4.27	−2	−46	62
	EC	**0.001**	**465**	**5.16**	**4.75**	**−22**	**−62**	**−40**
				4.76	4.43	−10	−60	−36
				4.37	4.10	−34	−56	−46

*Significant negative correlation between NT-proBNP and brain network centrality using the measures of global correlation (GCOR) and eigenvector centrality (EC). A significant negative correlation was obtained between NT-proBNP and both centrality measures in the same precuneus region as obtained with the group centrality differences (see Table 1). This negative correlation in the precuneus was obtained with both the initial (NT-proBNP(1)) and the follow-up measurement (NT-proBNP(2)). In addition, we obtained a significant negative correlation between NT-proBNP(2) and EC in cerebellar regions (see bottom rows). The table shows the *T*-maximum of each cluster (in bold) and two further local maxima more than 8 mm apart. pFWE = *P*-value with family-wise error correction at cluster-level; *K_E_* = cluster size in voxels; *x, y, z* = coordinates in mm.

We also looked for a potential positive relationship between brain network centrality and the cardiac biomarker NT-proBNP; however, we did not obtain any significant positive correlation for either centrality measure (GCOR and EC). This was the case for both the initial and the follow-up measurement of NT-proBNP.

We re-calculated all GCOR correlation analyses with variations of pre-processing and analysis parameters in order to check the robustness of our findings. These robustness analyses showed subtle deviations from the original results; thus, all robustness analyses revealed a significant positive correlation between GCOR and the initial LVEF, and a significant negative correlation between GCOR and both measurements of NT-proBNP. All results were obtained in the same anatomical location, namely in the precuneus (see Supplementary Table 4 for using band-pass filtering with a cut-off frequency of 0.01 Hz instead of 0.025 Hz; see Supplementary Table 5 for using the Friston24 model instead of the six motion parameters; and see Supplementary Table 6 using additional nuisance covariates in order to account for smoking, diabetes mellitus, systolic and diastolic blood pressure, Fazekas score and total intracranial volume).

### Seed-based correlation analysis

**Figure 4:**
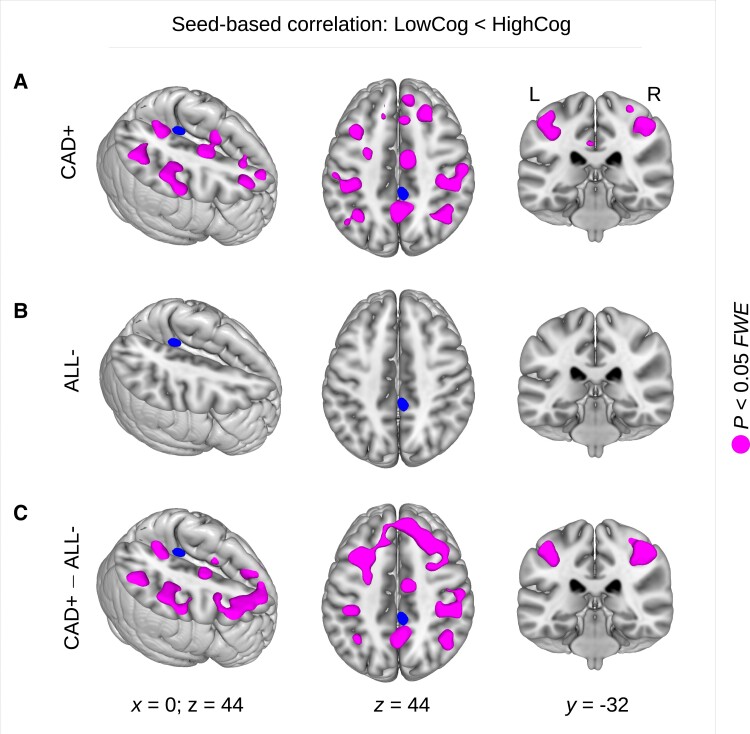
**Brain connectivity decrease in patients showing a reduced cognitive performance with heart failure.** (**A**) In patients with coronary artery disease and heart failure (CAD+), a seed-based connectivity (SBC) analysis revealed connectivity differences between patients with higher (HighCog) and lower (LowCog) cognitive performance. Diminished cognitive performance was found to be associated with a reduced brain connectivity between the precuneus and wide cortical areas including the motor system but also further frontal and parietal brain regions. (**B**) No SBC differences were found between HighCog and LowCog patients without heart failure (ALL−). (**C**) A significant interaction was obtained between the factors of heart failure (CAD+/ALL−) and cognition (HighCog/LowCog) showing diminished SBC with lower cognitive performance. Statistical analysis was performed using a general linear model with a full-factorial design implementing an interaction of both factors heart failure and cognitive performance. Significant results were obtained using a *t*-contrast with *P* < 0.05 using family-wise error (FWE) correction at cluster-level (see regions in magenta). L = left; R = right; *x, y, z* = coordinates in mm.

To further investigate seed-based connectivity between precuneus and the rest of the brain, individual seed-based correlation maps were analysed using a full-factorial design with the factors HF (CAD+ versus ALL−) and cognitive performance (above versus below average). We obtained a significant interaction between the factors HF and cognitive performance showing lower precuneus connectivity associated with cognitive performance below average in HF (*P* < 0.05, FWE-corrected; Fig. 4C).

*Post hoc* analyses were subsequently performed in order to investigate a potential relationship between precuneus connectivity and cognition within CAD+ and ALL− groups separately. In the CAD+ group, but not in the ALL− group, we found significant seed-based connectivity differences between higher and lower cognitive performance where lower precuneus connectivity was associated with cognitive performance below average (*P* < 0.05, FWE-corrected; Fig. 4A and B). In particular, this result suggests a specific HF-related connectivity reduction, involving lower cognitive performance, between precuneus and widespread cortical areas including the motor system as well as frontal and parietal regions.

Note that we also looked for the opposite direction of relationship between precuneus connectivity and cognitive performance (i.e. greater connectivity with lower cognitive performance) in both groups (CAD+ and ALL−) separately. No significant results were observed.

### Statistical analysis including grey matter density

To further investigate a potential relationship between our connectivity findings and the GMD changes observed in a preceding study,^3^ all statistical analyses were re-computed using GMD as an additional covariate of no interest. Interestingly, we did not obtain any significant result with the centrality group comparisons when including GMD within the model, neither with GCOR nor with EC. In addition, we also did not obtain any significant correlation between centrality (GCOR, EC) and LVEF and NT-proBNP except a significant negative correlation between GCOR and the initial value of NT-proBNP (see Supplementary Table 7). Thus, most of the comparisons and the correlations between centrality and HF-related biomarkers were predominantly explained by GMD differences in the precuneus. In contrast to this observation, our finding about the relationship between seed-based precuneus connectivity and cognitive performance was not affected by including precuneus GMD in the model; thus, the result was even more pronounced showing even larger clusters across widely distributed cortical regions (compare Fig. 4 with Supplementary Fig. 1). Note that the result was also obtained when modelling cognitive performance as a continuous variable (mean percentile rank across all four cognitive domains) instead of using two categories of high and low cognitive performance (see Supplementary Fig. 2).

### Motion effects

The analysis of head motion during fMRI data acquisition yielded overall very subtle effects. The mean FD was <1 mm for all patients in all groups. The maximum FD was less than the nominal voxel size of 3 mm (except a single patient who showed a maximum FD of 4.1 mm). When disregarding the largest 5% of FD values, the maximum remaining FD was <1.55 mm. Moreover, we did not find any consistent differences in the motion parameters between the patient groups CAD+ versus CAD, CAD+ versus no-CAD and CAD− versus no-CAD (*P* > 0.3 for all group comparisons with mean FD and max FD).

## Discussion

Our investigations suggest the precuneus as the brain region mainly affected by HF, with the region becoming more decoupled from the rest of the brain. In particular, in both comparisons, between CAD+ and CAD− and between CAD+ and no-CAD, we found significantly weaker brain centrality within the precuneus with two different centrality measures (GCOR and EC). Centrality was associated with two HF-related parameters, LVEF and NT-proBNP. In both cases, a relationship was observed between these biomarkers and brain connectivity, particularly in the precuneus. Subsequent seed-based correlation analysis showed weaker precuneus connectivity to be related to lower cognitive performance in HF patients. Thus, our study suggests the precuneus as a rather crucial brain region showing a major HF-related connectivity decline.

The precuneus is one of the major hubs within the brain’s default mode network.^55^ Interestingly, however, there is not much known about the impact of HF on functional brain connectivity, particularly between regions within the default mode network including the precuneus. So far, only a few studies have addressed HF-related connectivity alterations using fMRI. Recently, Park *et al*. investigated HF patients with resting-state fMRI^56^ using a graph-theoretical approach with the Brain Connectivity Toolbox.^57^ In line with our findings, they observed decreased connectivity between the precuneus and other brain regions including the cerebellum and parietal regions. The decrease in precuneus connectivity was suggested to result from an abnormal network coordination from the posterior parietal cortex.^56^ Other resting-state fMRI studies including patients with cardiac arrest found a major role of the precuneus, showing an overall decreased connectivity in the default mode network.^58,59^ Compared with healthy controls, patients with cardiac arrest demonstrated a reduced local efficiency of the precuneus in context of information transfer.^58^ Our finding might be explained by similar mechanisms, leading to reduced precuneus connectivity in our group of patients with HF compared with patients without HF. Notably, the precuneus was also linked to cognitive function in another fMRI study on young adult subjects who had been surgically treated for congenital heart disease.^60^ Within patients, working memory-related alterations in precuneus activity were discussed in the context of disrupted neural systems.^60^ In line with this, we also found diminished connectivity between the precuneus and widespread cortical regions, particularly related to reduced cognitive performance in HF. In summary, precuneus dysconnectivity seems to be a key feature leading to impairment of cognition in HF.^5^

The understanding of HF-related changes in cerebral blood flow (CBF) is certainly essential in further investigating the neuropathophysiological mechanisms leading to cognitive decline associated with HF. The literature supports the hypothesis that HF-related cardiac dysfunction impacts CBF, which in turn is consecutively related to cognitive function.^4,61,62^ A possible underlying mechanism is a chronic hypoperfusion in major brain regions, thus gradually leading to local tissue injury.^63^ Aside from a general decrease of global CBF with HF measured by radionuclide angiography,^64^ reduction in regional CBF has been detected in the precuneus using 99mTc-single-photon emission computed tomography in 17 patients with HF.^65^ Similar to these patients with HF, a decrease of regional CBF was found in the precuneus of 19 patients with AD using arterial spin-labelling,^66^ a finding confirmed in a meta-analysis.^27^ Our results might be linked to this CBF decrease by recent findings of Liang *et al*. showing a strong relationship between regional CBF and functional connectivity strength^67^ observed in the default mode network and, again, particularly in the precuneus. Recent results also suggest a linear relationship between CBF and network centrality.^68^ In turn, an HF-related decrease of CBF might explain our findings of reduced network centrality.

In CAD patients without HF, a voxel-wise regression analysis showed a relationship between cognitive decline and shorter arterial transit time (ATT) in the precuneus region,^69^ which was mainly attributed to CAD-related changes in the vessel wall.^70^ Specifically, this ATT change might be associated with altered arterial pulsatility due to a CAD-related loss of compliance in the form of arterial elasticity.^71,72^ In line with this hypothesis, loss of compliance can be further accompanied by lower CBF, which has already been observed with normal or ‘physiological’ aging, particularly in the precuneus.^73^ Furthermore, diminished brain connectivity has also been described in normal aging,^74–76^ including in the precuneus.^77,78^ Taken together, the sensitivity of the precuneus, along with normal aging process, might represent a link to our finding of a decrease of precuneus connectivity in patients with HF.

Precuneus dysconnectivity is also prevalent in AD, compared with that described in healthy aging.^74,79^ This is consistent with an AD-related decrease in cerebral glucose metabolism as shown by fluorodeoxyglucose [^18^F] positron emission tomography (PET), as well as increased amyloid deposition, measured by PET employing the Pittsburgh Compound-B amyloid tracer.^80^ Interestingly, there appears to be a link between precuneus hypometabolism and CAD as described in a fluorodeoxyglucose positron emission tomography study on cardiovascular risk factors based on the Framingham 10-year risk Coronary Heart Disease Risk Profile.^81^ Reduction in cerebral metabolism was detected in the precuneus when comparing a high-risk and a low-risk group. The authors discussed their finding in the context of pathologic brain changes similar to AD.^81^ Cerebral hypoperfusion is a well-known feature in AD, with the precuneus being the first affected region.^82,83^ This is also supported by a comprehensive meta-analysis in AD and its prediction.^27^ Our HF-related findings are located in exactly the same area of the precuneus (see Fig. 1). This suggests that structure is particularly sensitive to brain pathology and may represent a common source of functional deficit across conditions; however, to support this conclusion, further work should include AD biomarkers as PET with tau and/or amyloid tracers and quantitative measurements of cerebrospinal fluid tau and amyloid-beta species.

Beside the centrality group differences between patients with and without HF, we also found a relationship between centrality and HF-related measures LVEF and NT-proBNP across the whole sample of patients. In particular, we found a significant correlation between LVEF and both centrality measures in the precuneus. Although this analysis did not include any definition of HF (e.g. using an NT-proBNP cut-off), we obtained a centrality-LVEF-correlation in the same anatomical region as found with the group comparisons, namely in the precuneus, that suggests the LVEF as a biomarker for HF-related centrality decline. Similarly, we observed a negative correlation between precuneus centrality and NT-proBNP with both the initial and the follow-up measurement of NT-proBNP. Interestingly, both markers LVEF and NT-proBNP were studied in context of an HF-related reduction of CBF in elderly males.^62^ Reduced CBF was associated with reduced LVEF and with increased NT-proBNP levels. Thus, our findings of lower precuneus centrality might be related to the HF-associated reductions of CBF reflected by the biomarkers LVEF and NT-proBNP. Notably lower LVEF was also found with lower cognitive performance^84^ that might be related to reduced CBF and diminished precuneus centrality. Our finding might provide a potential link showing lower precuneus centrality with reduced cognitive performance in HF (see Fig. 4).

Remarkably, our present findings of network centrality decrease associated with HF run parallel to the structural brain changes, also predominantly in the precuneus, that we have reported previously.^3^ In the mentioned study, we performed structural MRI and voxel-based morphometry in the very same patient cohort. We found diminished GMD in the precuneus and other cortical regions such as the anterior and the posterior cingulate cortex (see Figs 1 and 3, colour-coded in green). In line with our findings, HF-related GMD reduction has also been described by Almeida *et al*. in the same regions, including the cingulate cortex and precuneus.^85^ Thus, HF-related diminished network centrality might be related to structural brain damage caused by HF. Indeed, our analysis with adjustment for changes in grey matter density confirmed the relationship between seed-based precuneus connectivity and cognitive performance, whereas group differences and correlation analysis results with biomarkers—here LVEF and NT-proBNP—mainly disappeared. One might conclude that, in our data, structural and functional changes explain an independent portion of the variance, where structural parameters are predominantly related to biomarkers of HF, i.e. LVEF and NT-proBNP, and functional connectivity parameters to cognitive performance.

The aforementioned hypothesis is supported by recent findings showing joint reduction of precuneus GMD and functional connectivity in MCI, a risk state for AD.^86^ In contrast, Qian *et al*. described a positive coupling of structural and functional degeneration specific to AD that was not present in MCI.^87^ Interestingly, the precuneus was shown to be involved in the conversion of these patients from MCI to AD.^27^ Thus, decline in both brain structure and connectivity might reflect the transition from MCI to AD.^87^ Similar to these findings, our data show a decline in both brain structure and connectivity, particularly in the precuneus. In addition, we found a link to cognitive performance, mainly in connectivity. Significant decrease of brain connectivity between precuneus and widespread cortical regions was found particularly in HF patients showing lower cognitive performance. This finding is in line with recent work showing HF-related brain injury, with cognitive decline, associated with reduced mean diffusivity.^88^ Taken together, these alterations in brain structure and connectivity might explain the overall decline in cognitive function in patients with HF.^5^

At this point, it is worth to mention, that physiological noise (heartbeat, respiratory cycle, blood pressure fluctuations) can lead to spurious signal contribution in the fMRI signal unless appropriate noise elimination techniques (e.g. band-pass filtering) are adopted.^89^ In our study, in patients presenting with coronary heart disease, the brain might be affected due to one or more out of several above-listed to ‘vascular risk factors’ as well as due to age-related structural changes of cerebral tissue which often correlate to a various extent. At the same time, however, these could represent potential confounders in functional imaging of the brain that relies on the BOLD signal, i.e. the amplitude and shape of the neurovascular response.^90^ We have no data available whether, and if so to what extent patients in our study have focal or global or vasculopathic changes (e.g. MR time-of-flight angiography). Nevertheless, in order to check for over- or underestimation in centrality measures, we examined a potential contribution of different co-existing diseases/risk factors or specific clinical measures to the resting-state BOLD fMRI signal by calculating all GCOR group analyses as well as GCOR correlation analyses with respective ‘nuisance’ variables; these were: systolic and diastolic arterial blood pressure (hypertension), diabetes mellitus, nicotine abuse and also the Fazekas score^54^ for assessment of micro-angiopathic lacunar lesion up to leukoencephalopathy (as a rough estimate for already structurally affected white matter), none of which exerted a significant impact on group comparison centrality maps. Thus, after all, the validity of the decreased centrality within the precuneus is supported by a decrease of precuneus grey matter density we had reported previously as converging results.

## Conclusion

The results presented in this study show an HF-related decline of brain connectivity between the precuneus and widely distributed cortical areas including the motor system but also further frontal and parietal regions. These effects were particularly found in patients with reduced cognitive performance. This connectivity decline was associated with two HF-biomarkers, namely, LVEF and NT-proBNP. Similar to current AD models showing precuneus connectivity decline as a key feature of brain degeneration, there might be common underlying mechanisms leading to brain connectivity alterations in HF and AD. This hypothesis would also be supported by specific network centrality alterations between precuneus and cortical regions particularly in patients with HF showing reduced cognitive performance. However, further work is necessary to elucidate potential common mechanisms of brain degeneration in HF and AD. To address this question, we suggest a combined assessment of different imaging modalities which includes: (i) structural MRI; (ii) task-free functional connectivity but also simple task-engaging fMRI to identify common network-relevant regions that in turn serve as seeds for (iii) fibre tracking on the basis of diffusion tensor imaging and (iv) histopathological surrogate markers for AD, such as amyloid and tau from cerebrospinal fluid or PET. We further suggest using a longitudinal approach in order to obtain an integrated view of the pathology in HF-associated neuropsychological as well as neuropsychiatric disorders. We believe such an approach will contribute to the understanding of AD pathology.

## Supplementary Material

fcad103_Supplementary_Data

## Data Availability

Data sets analysed during the current study are available on reasonable request. All data have been anonymized. fMRI data will be available in pre-processed fashion in the NIfTI format without any personal metadata. Individual brain connectivity maps and subsequent statistical analyses using SPM12 are publicly available in the Mendeley Data repository ‘Brain dysconnectivity with heart failure: Centrality and seed-based correlation maps obtained with functional MRI’ https://doi.org/10.17632/6h782nrb24.1

## Abbreviations

AD =: Alzheimer’s disease
ALL− =: group of patients showing no heart failure
ATT =: arterial transit time
BMI =: body mass index
BOLD =: blood oxygenation level dependent
CAD =: coronary artery disease
CAD+ =: group of patients showing coronary artery disease with heart failure
CAD− =: group of patients showing coronary artery disease but without heart failure
CBF =: cerebral blood flow
EC =: eigenvector centrality
EPI =: echo-planar imaging
FD =: frame-wise displacement
fMRI =: functional magnetic resonance imaging
FWE =: family-wise error
GCOR =: global correlation
GMD =: grey matter density
HF =: heart failure
LVEF =: left ventricular ejection fraction
MCI =: mild cognitive impairment
MPRAGE =: magnetization-prepared rapid gradient-echo
MRI =: magnetic resonance imaging
no-CAD =: group of patients without coronary artery disease
NT-proBNP =: N-terminal fragment of the pro-hormone brain-type natriuretic peptide
PET =: positron emission tomography
TE =: echo time
TR =: repetition time
VBM =: voxel-based morphometry
