# Supplementary material for: Brain dysconnectivity with heart failure
Source: Brain Commun. 2023 Mar 30;5(2):fcad103. doi: 10.1093/braincomms/fcad103 (PMC10116577; doi:10.1093/braincomms/fcad103)
Supplement: fcad103_Supplementary_Data [file fcad103_supplementary_data.pdf]

Supplementary Table 1. Brain network centrality decrease with heart failure*

	pFWE	K_E	*F/T*	*Z*	*x*	*y*	*z*
**GROUP**	**0.003**	**293**	**16.44**	**4.68**	**4**	**-38**	**52**
			12.83	4.13	-8	-34	48
			9.62	3.53	-6	-26	52
**CAD+ < CAD-**	**0.003**	**411**	**4.68**	**4.35**	**-8**	**-34**	**48**
			4.65	4.32	4	-40	54
			4.29	4.02	4	-30	50
**CAD+ < no-CAD**	**0.004**	**382**	**5.00**	**4.60**	**4**	**-38**	**52**
			4.38	4.10	-6	-26	52
			3.87	3.67	10	-26	48
**CAD+ < ALL-**	**<0.001**	**652**	**5.72**	**5.16**	**4**	**-38**	**52**
			4.99	4.59	-8	-34	48
			3.99	3.77	-8	-44	50

*Significant global correlation (GCOR) differences obtained with the same analysis shown in Table 1 except using temporal filtering with a cutoff frequency of **0.01Hz instead of 0.025Hz**.

Supplementary Table 2. Brain network centrality decrease with heart failure*

	pFWE	K_E	*F/T*	*Z*	*x*	*y*	*z*
**GROUP**	**0.018**	**208**	**14.01**	**4.32**	**4**	**-30**	**50**
			11.56	3.91	-8	-34	48
			10.00	3.61	16	-40	50
**CAD+ < CAD-**	**0.013**	**300**	**4.54**	**4.23**	**-8**	**-34**	**48**
			4.47	4.18	4	-30	50
			4.44	4.15	16	-40	50
**CAD+ < no-CAD**	**0.044**	**220**	**4.44**	**4.15**	**4**	**-30**	**52**
			4.41	4.13	8	-26	48
			4.30	4.03	-6	-26	52
**CAD+ < ALL-**	**0.002**	**420**	**5.29**	**4.83**	**4**	**-30**	**50**
			4.90	4.52	6	-36	52
			4.67	4.34	-8	-34	48

*Significant global correlation (GCOR) differences obtained with the same analysis shown in Table 1 except using nuisance regression with **the Friston24 model instead of the six motion parameters**.

Supplementary Table 3. Brain network centrality decrease with heart failure*

	pFWE	K_E	*F/T*	*Z*	*x*	*y*	*z*
**GROUP**	**0.001**	**348**	**15.61**	**4.52**	**4**	**-38**	**52**
			13.40	4.19	-6	-34	48
			10.76	3.73	-12	-30	44
**CAD+ < CAD-**	**<0.001**	**554**	**4.79**	**4.41**	**-6**	**-34**	**48**
			4.61	4.26	4	-38	52
			4.53	4.20	16	-40	50
**CAD+ < no-CAD**	**0.006**	**336**	**4.82**	**4.43**	**4**	**-36**	**52**
			4.15	3.89	10	-26	46
			4.05	3.80	-6	-26	52
**CAD+ < ALL-**	**<0.001**	**754**	**5.58**	**5.01**	**4**	**-38**	**52**
			5.09	4.64	-6	-34	48
			4.00	3.76	14	-34	44

*Significant global correlation (GCOR) differences obtained with the same analysis shown in Table 1 **including additional nuisance covariates in order to account for smoking, diabetes mellitus, systolic and diastolic blood pressure, the Fazekas score, and the total intracranial volume**.

Supplementary Table 4. Correlation between brain network centrality and heart failure related biomarkers across all patients*

	pFWE	K_E	*T*	*Z*	*x*	*y*	*z*
**LVEF(1)**	**<0.001**	**787**	**5.27**	**4.84**	**6**	**-30**	**52**
			4.29	4.04	-6	-44	50
			4.23	3.99	10	-44	56
**NT-proBNP(1)**	**<0.001**	**690**	**5.97**	**5.37**	**4**	**-38**	**52**
			4.61	4.30	-8	-34	48
			3.75	3.57	-10	-44	50
**NT-proBNP(2)**	**0.001**	**520**	**4.43**	**4.16**	**-2**	**-42**	**56**
			4.08	3.86	8	-38	46
			3.94	3.74	-8	-48	74

*Significant positive correlation between brain network centrality (obtained by global correlation, GCOR) and the initial measurement of the left ventricular ejection fraction (LVEF) obtained with the same analysis as shown in Table 2, and significant negative correlation between GCOR and both NT-proBNP values as shown in Table 3, except using temporal filtering with a cutoff frequency of **0.01Hz instead of 0.025Hz**.

Supplementary Table 5. Correlation between brain network centrality and heart failure related biomarkers across all patients*

	pFWE	K_E	*T*	*Z*	*x*	*y*	*z*
**LVEF(1)**	**0.001**	**485**	**4.92**	**4.56**	**6**	**-30**	**52**
			4.42	4.14	8	-44	56
			3.98	3.77	-6	-32	50
**NT-proBNP(1)**	**0.001**	**540**	**5.65**	**5.13**	**4**	**-36**	**52**
**NT-proBNP(2)**	**0.012**	**297**	**3.86**	**3.67**	**8**	**-38**	**48**
			3.79	3.61	-10	-46	72
			3.74	3.57	0	-36	54

*Significant positive correlation between brain network centrality (obtained by global correlation, GCOR) and the initial measurement of the left ventricular ejection fraction (LVEF) obtained with the same analysis as shown in Table 2, and significant negative correlation between GCOR and both NT-proBNP values as shown in Table 3, except using nuisance regression with **the Friston24 model instead of the six motion parameters**.

Supplementary Table 6. Correlation between brain network centrality and heart failure related biomarkers across all patients*

	pFWE	K_E	*T*	*Z*	*x*	*y*	*z*
**LVEF(1)**	**<0.001**	**995**	**4.62**	**4.29**	**-8**	**-44**	**48**
			4.62	4.29	6	-30	52
			4.52	4.21	-4	-36	50
**NT-proBNP(1)**	**<0.001**	**876**	**6.24**	**5.52**	**4**	**-38**	**52**
			4.90	4.51	14	-38	50
**NT-proBNP(2)**	**0.001**	**454**	**4.24**	**3.97**	**0**	**-38**	**50**
			4.11	3.87	8	-38	46

*Significant positive correlation between brain network centrality (obtained by global correlation, GCOR) and the initial measurement of the left ventricular ejection fraction (LVEF) obtained with the same analysis as shown in Table 2, and significant negative correlation between GCOR and both NT-proBNP values as shown in Table 3, **including additional nuisance covariates in order to account for smoking, diabetes mellitus, systolic and diastolic blood pressure, the Fazekas score, and the total intracranial volume**.

Supplementary Table 7. Correlation between brain network centrality and the initial measurement of NT-proBNP across all patients*

	pFWE	K_E	*T*	*Z*	*x*	*y*	*z*
**NT-proBNP(1)**	**0.016**	**272**	**4.80**	**4.46**	**4**	**-38**	**52**
			4.05	3.83	-6	-36	48
			3.73	3.55	16	-40	50

*Significant negative correlation between global correlation (GCOR) and the initial measurement of NT-proBNP(1) as shown in Table 3, except including **the precuneus gray matter density (GMD) as an additional nuisance covariate** in the statistical analysis.

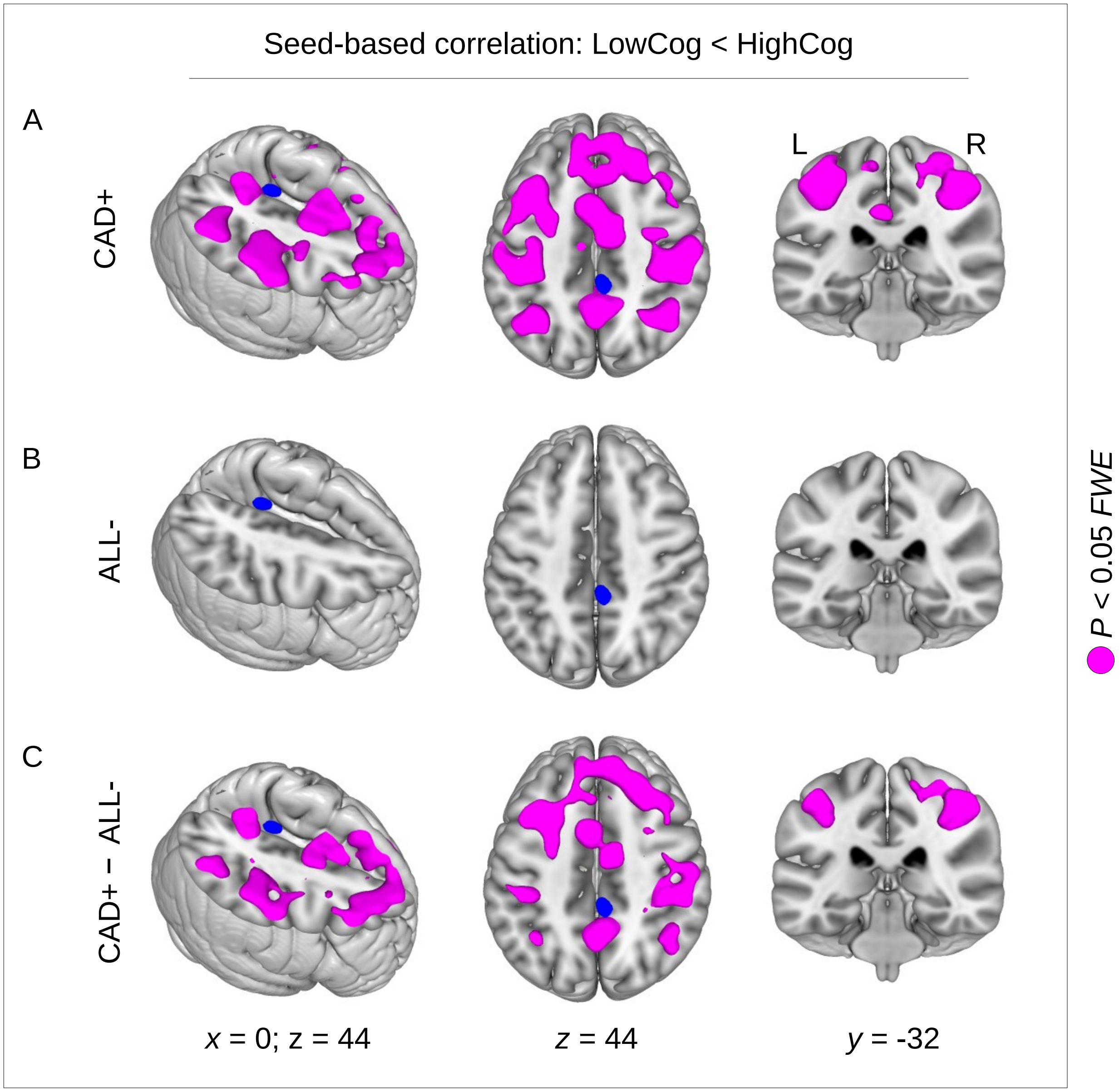

Supplementary Figure 1. Brain connectivity decrease in patients showing a reduced cognitive performance with heart failure. This figure shows the same content as Figure 4, except including **the precuneus gray matter density (GMD) as an additional nuisance covariate** in the statistical analysis.

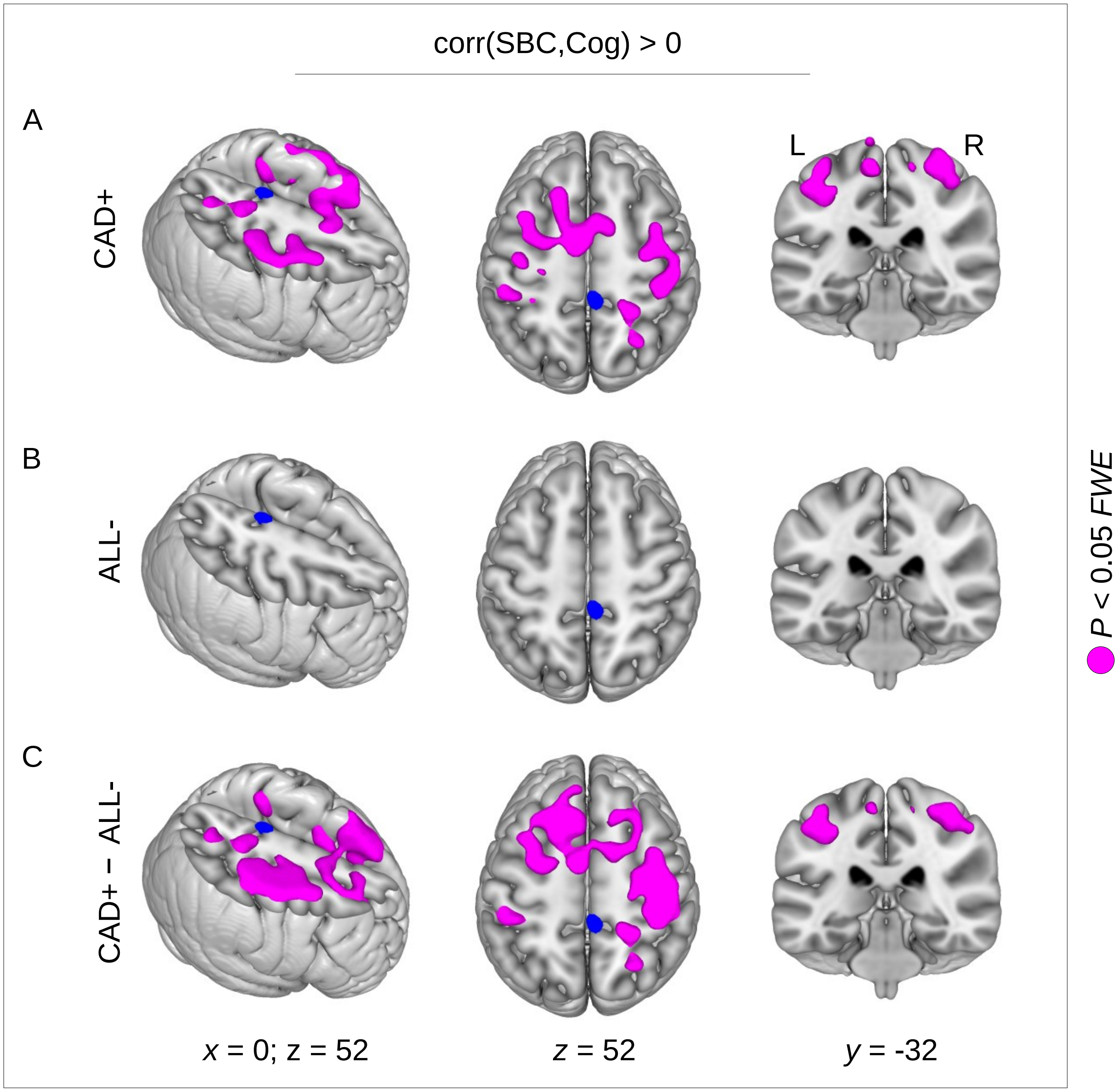

Supplementary Figure 2. Brain connectivity decrease in patients showing a reduced cognitive performance with heart failure. This figure shows the same content as Figure 4, except **modeling cognitive performance as a continuous variable (mean percentile rank across all four cognitive domains) and including the precuneus gray matter density (GMD) as an additional nuisance covariate in the statistical analysis.**
